# An Outbreak of COVID-19 Associated with a Recreational Hockey Game — Florida, June 2020

**DOI:** 10.15585/mmwr.mm6941a4

**Published:** 2020-10-16

**Authors:** David Atrubin, Michael Wiese, Becky Bohinc

**Affiliations:** ^1^Florida Department of Health; ^2^Florida Department of Health, Hillsborough County; ^3^Florida Department of Health, Pinellas County.

On June 16, 2020, a recreational ice hockey game was played at an ice rink in the Tampa Bay, Florida, metropolitan area. Teams A and B, each consisting of 11 players (typically six on the ice and five on the bench at any given time), included men aged 19–53 years. During the 5 days after the game, 15 persons (14 of the 22 players and a rink staff member) experienced signs and symptoms compatible with coronavirus disease 2019 (COVID-19)*; 13 of the 15 ill persons had positive laboratory test results indicating infection with SARS-CoV-2, the virus that causes COVID-19. Widespread transmission of SARS-CoV-2 has been documented at a choir practice (*1*) and at meat processing plants (*2*,*3*); however, apart from an outbreak involving 57 infected dancers that has been linked to high-intensity fitness dance classes in South Korea (*4*) and a cluster of five infected persons at a squash facility in Slovenia (5), few published reports are available regarding transmission associated with specific sports games or practices. In addition, outbreaks of COVID-19 infections among amateur hockey players in the United States have recently been reported in the news.^†^

On June 19, 2020, the Florida Department of Health was notified of a team A player (the index patient) who experienced fever, cough, sore throat, and a headache beginning on June 17, the day after he had participated in an evening game; 2 days later, a nasal specimen was obtained, which tested positive for SARS-CoV-2 by Sofia SARS Antigen Fluorescent Immunoassay (https://www.quidel.com/immunoassays/coronavirus). An investigation by the Florida Department of Health revealed that eight of 10 team A players (excluding the index patient), five of 11 players from team B, and one rink staff member experienced COVID-19 signs and symptoms during June 18–21 (Figure), 2–5 days after the game. Excluding the index patient, 13 of the 21 (62%) players experienced illness. Among the 15 total cases in this outbreak, 11 patients had positive SARS-CoV-2 reverse transcription–polymerase chain reaction results, two had positive antigen tests,^§^ and two were not tested.^¶^ Asymptomatic players did not seek testing. Neither of the two on-ice referees experienced symptoms. Because the investigation was deemed public health practice, approval by the Florida Department of Health Institutional Review Board was not required.

**FIGURE:**
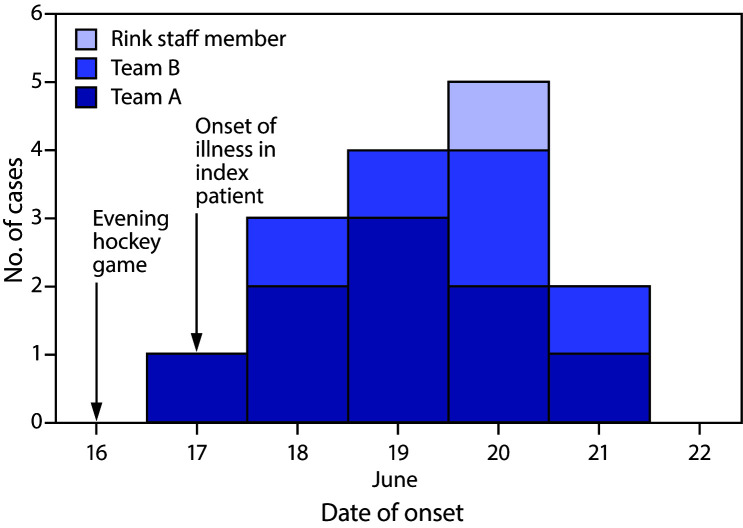
COVID-19 cases associated with a recreational ice hockey game, by date of onset (N = 15) — Florida, June 2020 **Abbreviation:** COVID-19 = coronavirus disease 2019.

Ice hockey involves vigorous physical exertion accompanied by deep, heavy respiration, and during the game, players frequently move from the ice surface to the bench while still breathing heavily. In this game, hockey-specific face protection varied and included metal cages or plastic half-shields (covering the eyes and the upper part of the nose); some players do not wear face protection. Cloth face masks for disease control were not used in the locker rooms or during the game. A standard ice rink in the United States measures 200 feet (61 meters) by 85 feet (26 meters). Boards and plexiglass, extending upward to approximately 10 feet (3 meters), surround the ice surface creating a physically segregated playing area. In addition to the 60-minute game time on the ice, during which players frequently came within 6 feet of one another, each team used a separate locker room, typically for 20 minutes before and after the game. Players from the teams did not have other common exposures in the week before the game. The median incubation period for SARS-CoV-2 is 4–5 days from exposure to symptom onset and ranges from 2–14 days.** Although more than one player might have been infectious during the game, it is hypothesized that the index patient was the source of SARS-CoV-2 transmission for the other players while he was presymptomatic.

The ice rink provides a venue that is likely well suited to COVID-19 transmission as an indoor environment where deep breathing occurs, and persons are in close proximity to one another. An Italian study estimating the rate of SARS-CoV-2 emission by infectious persons based on viral load in the mouth showed that during heavy exercise, a high viral emission rate can be reached during oral breathing (*6*). The higher proportion of infected players on the index patient’s team might result from additional exposures to the index patient in the locker room and on the player bench, where players sit close to one another.

A limitation of this investigation was that not all players from the game sought testing, and asymptomatic infections were possibly not identified. The indoor space and close contact between players during a hockey game increase infection risk for players and create potential for a superspreader event, especially with ongoing community COVID-19 transmission. Superspreader events, in which one infectious person infects many others, can lead to explosive growth at the beginning of an outbreak and facilitate sustained transmission later in an outbreak (*7*). This game involved a relatively limited number of players and only one spectator, who remained symptom-free and was not tested (the limited number of spectators was not related to rink policy); however, hockey games can include up to 20 players on each of the two teams and many spectators in the arena.

The high proportion of infections that occurred in this outbreak provides evidence for SARS-CoV-2 transmission during an indoor sporting activity where intense physical activity is occurring. In response, Florida Department of Health staff members provided isolation and quarantine recommendations to the persons in the rink during the game and advised ice rink management on COVID-19 risk and disease control.

## Footnotes

* Signs and symptoms included fever, myalgia, cough, sore throat, headache, and loss of sense of taste or smell.

† https://patch.com/new-jersey/middletown-nj/cluster-covid-cases-teams-practiced-middletown-rink. https://www.ajc.com/news/junior-hockey-league-held-likely-covid-19-spreader-event-in-cobb/EID5ZRFLMRGGFCZ7NBK7BV4S5A/.

§ Information on the type of antigen test used for the second patient was not available.

¶ Whether patients were tested and what type of testing was performed was determined by the health care providers who evaluated the players.

** https://www.cdc.gov/coronavirus/2019-ncov/symptoms-testing/symptoms.html.
